# Structural definition of polyspecific compensatory ligand recognition by P-glycoprotein

**DOI:** 10.1107/S2052252520005709

**Published:** 2020-06-06

**Authors:** Christina A. Le, Daniel S. Harvey, Stephen G. Aller

**Affiliations:** a Department of Pharmacology and Toxicology, University of Alabama at Birmingham, Birmingham, AL 35294, USA

**Keywords:** P-glycoprotein, polyspecificity, crystallography, multidrug resistance

## Abstract

The multidrug transporter P-glycoprotein (Pgp)/ABCB1/MDR1 plays an important role in multidrug resistance (MDR) and detoxification owing to its ability to efflux an unusually large and chemically diverse set of substrates. Previous phenylalanine-to-alanine scanning mutagenesis of Pgp revealed that nearly all mutations retained full MDR function and still permitted substrate transport. This suggests that either the loss of any single aromatic side chain did not affect the ligand-binding modes or that highly adaptive and compensatory drug recognition is an intrinsic property including ligand-binding shifts that preserve function. To explore this hypothesis, the ATPase function and crystallographic localization of five single-site mutations in which the native aromatic residue directly interacted with the environmental pollutant BDE-100, as shown in previous crystal structures, were tested. Two mutants, Y303A and Y306A, showed strong BDE-100 occupancy at the original site (site 1), but also revealed a novel site 2 located on the opposing pseudo-symmetric half of the drug-binding pocket (DBP). Surprisingly, the F724A mutant structure had no detectable binding in site 1 but exhibited a novel site shifted 11 Å from site 1. ATPase studies revealed shifts in ATPase kinetics for the five mutants, but otherwise indicated a catalytically active transporter that was inhibited by BDE-100, similar to wild-type Pgp. These results emphasize a high degree of compensatory drug recognition in Pgp that is made possible by aromatic amino-acid side chains concentrated in the DBP. Compensatory recognition forms the underpinning of polyspecific drug transport, but also highlights the challenges associated with the design of therapeutics that evade efflux altogether.

## Abstract 2

Polyspecific ligand recognition by P-glycoprotein includes compensatory mechanisms in the form of ligand-binding shifts when ligand-interacting residues are challenged by mutagenesis. Point mutations of ligand-interacting phenylalanine residues to alanines resulted in binding shifts of an environmental pollutant, yet its inhibitory function on ATPase activity was preserved.

## 1. Introduction   

P-glycoprotein (Pgp), a member of the ATP-binding cassette (ABC) superfamily, is one of the most promiscuous drug transporters in nature and is capable of binding and expelling a very large number of substrates from the cell (Ambudkar *et al.*, 1999 ▸; Schinkel & Jonker, 2003 ▸). Over time, cancer cells can develop resistance to a wide range of compounds by the upregulation of Pgp expression (Abdallah *et al.*, 2015 ▸; Gottesman & Ling, 2006 ▸; Roninson *et al.*, 1984 ▸, 1986 ▸; Shen *et al.*, 1986 ▸; Ueda *et al.*, 1986 ▸). Pgp therefore represents a major barrier to effective cancer chemotherapy since it enables cancer cells to develop resistance to several cytotoxic drugs (Szakács *et al.*, 2006 ▸), yet the mechanisms by which Pgp recognizes many substrates, a concept known as polyspecificity, are not well understood. An induced-fit model has been proposed that involves changes in the relative positions of transmembrane segments that adapt to allow the binding of particular substrates (Loo *et al.*, 2003 ▸). The opening–closing motion of Pgp is thought to alter the surface topology of the internal drug-binding pocket (DBP), accommodating the binding of different substrates (Esser *et al.*, 2017 ▸). These biochemical and structural approaches, among many others, point to relatively large changes in the conformation and dynamics of Pgp as a mechanism for its polyspecific substrate recognition. We wondered whether more subtle changes to the DBP of Pgp might also comprise a mechanism for its polyspecificity. A structural study of ligand shifts in response to a challenge to the binding capacity of the DBP by site-directed mutagenesis might be an effective approach to test this hypothesis and has not been undertaken previously.

Several structures of Pgp determined by X-ray crystallo­graphy (XRC) and cryo-EM revealed small molecules bound to the DPB (Nicklisch *et al.*, 2016 ▸; Szewczyk *et al.*, 2015 ▸; Alam *et al.*, 2019 ▸; Aller *et al.*, 2009 ▸). The structures highlight a high concentration of aromatic residues in the transmembrane α-helices that form the DBP, many of which are directly engaged in contacts with ligands. The abundance of aromatic residues potentially allows aromatic interactions (π–π, cation–π and *X*H–π) between the transporter and ligands throughout the DBP. Thus, it appears that aromaticity is likely to play a role in ligand/substrate recognition, *i.e.* specificity, but could also permit many different substrates to be recognized and transported, *i.e.* polyspecificity.

The binding mode of the environmental pollutant BDE-100 in the DBP of Pgp was also previously captured by XRC and it was shown to be a potent inhibitor of verapamil-stimulated ATPase activity of the transporter (Nicklisch *et al.*, 2016 ▸). BDE-100 serves as a robust molecular probe owing to its simplified structure that lacks rotatable bonds and contains five Br atoms that allow sensitive anomalous difference Fourier electron-density localization by XRC. Based on the mouse Pgp–BDE-100 co-crystal structure (PDB entry 4xwk), we selected five aromatic residues (Tyr303, Tyr306, Phe724, Phe728 and Phe979) that appeared to form direct contacts with BDE-100 for mutagenesis to test the hypothesis of polyspecificity compensation in the form of shifts in ligand-binding modes. Individual aromatic residues were mutated to alanines on a background wild-type mouse Pgp mimetic mutation, C952A, that had also previously been shown to form well diffracting crystals (Aller *et al.*, 2009 ▸). The ATPase activity and crystallographic localization of BDE-100 in the Pgp mutants were compared with those of a parent C952A mutant control as a basis for measuring changes in Pgp function and shifts in ligand-binding modes, respectively. To our surprise, two mutants, Y303A and Y306A, preserved BDE-100 binding to the original site (site 1) but also allowed strong binding to a second distal site in the DBP (site 2). BDE-100 binding to the F979A mutant was indistinguishable from that to the parent C952A mutant, but the F724A mutant lost BDE-100 binding at site 1 and gained binding at a third site (site 3) that was formed at the pseudosymmetric axis of the DBP. Verapamil-stimulated ATPase activity was robust in four of the five mutants, which is consistent with the previous finding that each corresponding mutant in human Pgp retained full MDR function in live cells (Loo & Clarke, 1993 ▸). Furthermore, in four mutants BDE-100 inhibited verapamil-stimulated ATPase activity, suggesting that its basic functional interaction with Pgp was preserved despite the shifts to new binding sites.

Our results offer a structural definition of Pgp polyspecificity, including a compensatory mechanism of ligand recognition, which remains intact even when specific aromatic interactions with the ligand are eliminated. The results also highlight the difficulties associated with designing inhibitors of Pgp-mediated transport, but may illuminate more productive efforts at designing therapeutics with improved bioavailability and better pharmacokinetics that can evade recognition and transport by Pgp.

## 2. Experimental procedures   

### 2.1. Pgp expression and purification   

P-glycoprotein and mutant constructs on a C952A template were overexpressed in *Pichia pastoris* with minimal glycerol. The method of expression and purification of mouse Pgp in *P. pastoris* has been described previously (Aller *et al.*, 2009 ▸; Li *et al.*, 2014 ▸). After transforming *P. pastoris* with mouse Pgp and a C-terminal 6×His tag, the cells were grown as a 16 l culture in a Bioflow 415 bioreactor (New Brunswick Scientific). Following overnight methanol induction (0.5 ml min^−1^), the cells were harvested and lysed at 276 MPa by a single pass through a cell disrupter (TS-Series; Constant Systems). Cell debris was separated by centrifugation at 15 900*g* at 4°C. Membranes were isolated by centrifugation at 38 400*g* at 4°C for 90 min and resuspended in 750 ml 75 m*M* NaCl, 15% glycerol, 20 m*M* Tris–HCl pH 8.0. The Pgp extracted from solubilized membranes with 9% Triton X-100 was purified using nickel–nitrilotriacetic acid (Ni–NTA) Superflow resin (Qiagen) on an ÄKTA FPLC. The Ni–NTA eluate was concentrated to 1 ml (Centricon YM-100; Millipore) and ultracentrifuged for 1.5 h, and the supernatant was loaded onto a size-exclusion column (Superdex 200 16/60; GE Healthcare) that had been pre-equilibrated in SEC buffer [10 m*M* HEPES pH 7.5, 75 m*M* NaCl, 0.0675% dodecylmaltoside (DDM), 0.04% sodium cholate, 0.1 m*M* tris(2-carboxylethyl)phosphine (TCEP)]. Elution peaks from 55 to 65 min were pooled and the concentration was determined by the Coomassie Plus Protein Assay (Pierce) at 595 nm using a standard curve of bovine serum albumin.

### 2.2. ATPase activity   

The ATPase activity of purified Pgp was measured using an ATP-regenerating system at 37°C as described by Vogel & Steinhart (1976 ▸) and modified by Urbatsch *et al.* (1995 ▸). Briefly, 2 µg Pgp was added to 100 µl 50 m*M* Tris–HCl pH 7.5 buffer consisting of 10 m*M* Mg^2+^-ATP, 6 m*M* phosphoenol­pyruvate, 1 m*M* NADH, 10 units of lactate dehydrogenase, 10 units of pyruvate kinase and test compounds over a range of concentrations. ATP hydrolysis was determined by the decrease in NADH absorbance at OD_340_ using a SpectraMax Plus spectrophotometer and *SoftMax Pro* version 5.4.1 (Molecular Devices). The ATPase activity was calculated using the equation ΔOD/(∊ × [protein] × time), where ΔOD is the change in absorbance and ∊ is the molar extinction coefficient for NADH. EC_50_ values were calculated using *GraphPad Prism* over the entire concentration range.

### 2.3. Reductive methylation and crystallization of mouse Pgp   

To reproduce the binding of BDE-100 to Pgp and maintain crystal isomorphism, we utilized the reductive-methylation protocol of Ward *et al.* (2013 ▸) with some modifications. Fresh dimethylamine–borane (20 m*M*; Sigma–Aldrich) and 16% formaldehyde (40 m*M*; Thermo Scientific) were added to 1–3 mg mouse Pgp. The mixture was incubated for 2 h at 4°C and further dimethylamine–borane and formaldehyde were added after 2 h. After incubation for 16 h at 4°C, the reaction was quenched with cold glycine on ice for 2 h and flushed with SEC buffer (10 m*M* Tris–HCl pH 7.5, 75 m*M* NaCl, 0.0375% DDM, 0.04% sodium cholate, 0.1 m*M* TCEP). The eluted protein was concentrated to 2–3 mg ml^−1^ and incubated with 0.5 m*M* BDE-100 (AccuStandard) dissolved in DMSO in the dark for 16 h at 4°C. The precipitate was centrifuged and the extracted sample was diluted 1:10 in SEC buffer to give a final concentration of 10–13 mg ml^−1^. Crystallization was set up using 3 µl sitting drops in 24-well Cryschem plates (Hampton Research) with a 1:1 ratio of protein solution and reservoir solution [0.1 *M* HEPES, 50 m*M* lithium sulfate, 10 m*M* EDTA, 26–28.5%(*w*/*v*) polyethylene glycol (PEG) 600 pH 7.9–8.4]. Crystals could be visualized after ∼3 days at 4°C and reached full size in ∼4 weeks.

The crystals were cryoprotected using an identical pH and identical lithium sulfate and EDTA concentrations as the growth conditions but with 29–30% PEG.

### 2.4. Data collection and model refinement   

**Figure 1:**
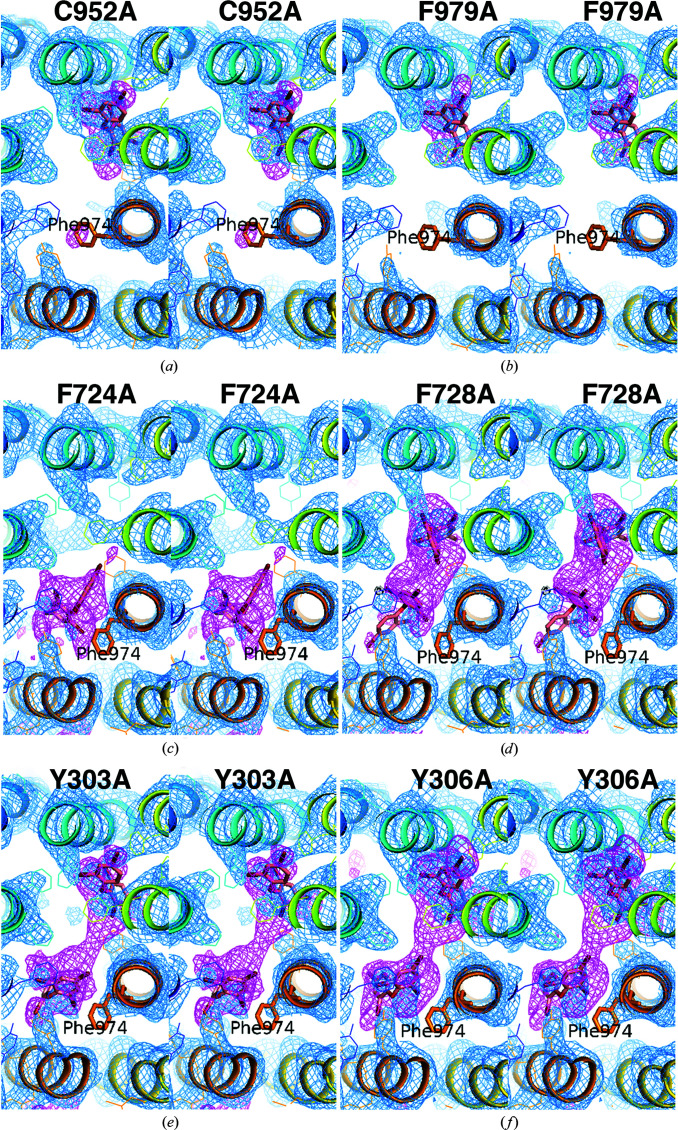
The electron density of six Pgp mutants and localization of BDE-100 by X-ray crystallography. Stereoviews are from the extracellular side perpendicular to the membrane. The blue mesh represents 2*mF*_o_ − *DF*_c_ density (*B* factor sharpened by 50–75 Å^2^) contoured to 1.0σ. The magenta mesh represents anomalous difference Fourier electron density from the Br atoms of BDE-100 using X-ray data collected at the bromine anomalous absorption peak (13.48 keV). Contour levels are as follows: 5.5σ for C952A, 5.0σ for F979A, 4.0σ for F724A, 3.8σ for F728A and 4.2σ for Y303A and Y306A. BDE-100 in each structure is shown in stick representation with Br atoms colored red. Phe974, which undergoes a disorder-to-order transition upon binding in site 2, is labeled in all six panels. The corresponding side views are shown in Supplementary Fig. S1.

X-ray diffraction data were collected at 100 K on beamline 23-ID-D at the Advanced Photon Source (APS). MAD scans were obtained from Pgp–BDE-100 co-crystals near the Br *K* edge to optimize for the strongest anomalous signal at the beginning of each data-collection session and whenever data were collected from a new mutant. The data were processed, integrated and scaled with *HKL*-2000 (Otwinowski & Minor, 1997 ▸). The Pgp structures were solved by molecular replacement using the previously solved BDE-100 complex structure (PDB entry 4xwk; Nicklisch *et al.*, 2016 ▸) without the BDE-100 (Table 1 ▸) using an initial rigid-body refinement of the two pseudosymmetric halves of the protein. Subsequent rounds included the full molecule with chains linked including ligands. *Phenix.elbow* was used to produce ligand description dictionaries (Adams *et al.*, 2010 ▸) and the placement of BDE-100 was verified by anomalous scattering of the Br atoms and OMIT density (see Fig. 1 ▸ for top views, Supplementary Fig. S1 for side views and Supplementary Fig. S2 for ligand *F*_o_ − *F*_c_ OMIT maps) calculated via *CNS* (Brünger *et al.*, 1998 ▸). The structures agreed with the previously solved structure as our control mutant (C952A) contained BDE-100 in the same position based on experimental data. All models were subjected to rigid-body,* xyz*, group *B*-factor and individual *B*-factor refinement using *phenix.refine* (Afonine *et al.*, 2010 ▸) and the model geometry was monitored using *MolProbity* (Chen *et al.*, 2010 ▸) in the absence of the ligand. The BDE-100 atoms were then modeled and the structures were subjected to the same refinement steps including occupancy refinement using an occupancy of 0.5 as the starting point. The occupancy refinement was justified on the basis that fixing the occupancy at 1.0 for our structures as well as for the deposited PDB entry 4xwk resulted in strongly negative (−4σ to −6σ) *mF*_o_ − *DF*_c_ electron density surrounding the ligands, which was still present after additional refinement steps using a fixed occupancy of 1.0. We conclude there is a sufficient data-to-parameter ratio to refine both *B* factors and occupancy for the BDE-100 ligand in PDB entry 4xwk as well as in all of our structures. Our own refinement of PDB entry 4xwk yielded a ligand occupancy of 0.66 and our control mutant C952A agreed very well, with a refined occupancy of 0.67 (for a full list, see Table 2 ▸). The refined occupancy values for ligands also agreed very well with the intensities of their respective anomalous difference Fourier electron densities. Data statistics and parameters supporting the quality of the structures are presented in Table 1 ▸ and Supplementary Table S1. Figures were prepared and r.m.s.d.s were calculated in *PyMOL* (version 1.8; Schrödinger). Atomic coordinates of the co-crystal structures of BDE-100 with the C952A Pgp control mutant, as well as of BDE-100 with the Pgp double mutants, were deposited in the Protein Data Bank with the following codes: 6ujn (C952A), 6ujt (C952A/Y303A), 6ujw (C952A/Y306A), 6ujr (C952A/F724A), 6ujs (C952A/F728A) and 6ujp (C952A/F979A).

## 3. Results and discussion   

Previous site-directed mutagenesis of Pgp/MDR1 coupled with cell-based and membrane-based biochemical assays showed that TM5, TM6, TM7 and TM12 contribute to the common binding site for the ATPase inhibitors QZ59-SSS and tariquidar, as well as the substrates cyclosporine A, valinomycin and 5′-fluorosulfonylbenzonyl 5′-adenosine (FSBA) (Chufan *et al.*, 2013 ▸, 2015 ▸). Mutations of Tyr307, Gln725 and Val982 in human Pgp to cysteine resulted in the loss of FSBA inhibition of Pgp labeling by the transport substrate [^125^I]-iodoarylazidoprazosin (Chufan *et al.*, 2013 ▸). Since the substrates (cyclosporine A, valinomycin and FSBA) and inhibitors (QZ59-SSS and tariquidar) retained their ATPase activity profile for the Pgp mutants, it was concluded that the substrates and inhibitors bound to alternate sites owing to mutation of the primary sites as a form of polyspecificity (Chufan *et al.*, 2013 ▸, 2015 ▸).

Structural studies have also identified amino-acid residues that directly interact with ligands and have even revealed multiple ligand-binding sites for several small molecules within the drug-binding pocket (DBP; Alam *et al.*, 2019 ▸; Szewczyk *et al.*, 2015 ▸). In the co-crystal structure of mouse Pgp with the environmental pollutant BDE-100 (PDB entry 4xwk), the BDE-100 ligand adopted a well defined single binding site in the DBP comprising the same transmembrane regions, *i.e.* TM5, TM6, TM7 and TM12, and appeared to make significant contact with the side chains of the aromatic residues Tyr303, Tyr306, Phe724, Phe728 and Phe979 (Nicklisch *et al.*, 2016 ▸). BDE-100 has also been shown to be a potent inhibitor of the verapamil-stimulated ATPase activity of Pgp. To test for the possibility of compensatory ligand recognition in Pgp in the form of ligand-binding shifts that still preserve the essential ligand effect on ATPase, we mutated these positions separately on the well diffracting C952A background (Aller *et al.*, 2009 ▸), measured the effect of BDE-100 on verapamil-stimulated ATPase activity and localized BDE-100 binding by X-ray crystallography (XRC).

The six crystal structures presented here reveal multiple binding modes of BDE-100 that depend on the mutation of a single contacting aromatic residue to alanine and the location of the mutant in the DBP. All structures were determined in the 3.98–4.17 Å resolution range and adopted the same inward-facing conformation as wild-type Pgp (Supplementary Fig. S3). Accurate BDE-100 localization and modeling was accomplished by inspecting the anomalous difference Fourier electron density of the Br atoms as well as difference Fourier (*mF*_o_ − *DF*_c_ and 2*mF*_o_ − *DF*_c_) electron density (Fig. 1 ▸). Anomalous peaks for multiple bromines were strong, and all Br atoms on BDE-100 in all of our structures were optimally visualized at contour levels between 3.8σ and 5.5σ.

Site 1 binding of BDE-100 (Nicklisch *et al.*, 2016 ▸; PDB entry 4xwk) was preserved in our C952A control (Supplementary Fig. S3) and was formed predominantly by the aromatic residues Tyr303, Tyr306, Phe310, Phe331, Phe724, Phe728, Phe755 and Phe979. Interestingly, the F979A mutant did not perturb BDE-100 binding to site 1 [Fig. 1 ▸(*b*)]. A close inspection shows that the Phe979 side chain in the other structures appears to be just outside van der Waals contact distance with the ligand (not shown), and therefore it does not appear to contribute much to the binding energy. Surprisingly, the F724A mutant exhibited no detectable BDE-100 occupancy in site 1, but revealed a novel site 3 that was shifted ∼11 Å from site 1 and was located directly on the axis of pseudosymmetry of the DBP formed by residues Met68, Phe71, Phe332, Phe728, Tyr949, Phe974 and Val978 [Fig. 1 ▸(*c*)]. The F728A, Y303A and Y306A mutants showed dual-occupancy BDE-100 binding in sites 1 and 2 [Figs. 1 ▸(*d*), 1 ▸(*e*) and 1 ▸(*f*)] and the ligands were in contact with the following residues: Met68, Phe71, Tyr303, Tyr306, Phe310, Phe331, Phe332, Gln721, Phe724, Ser725, Phe728, Val731, Phe755, Tyr949, Leu971, Phe974 and Phe979. The site 1 residues present in the Y303A and Y306A mutants but not the F728A mutant were Tyr303 or Tyr306 or Gln721, Ser725, Phe728 and Val731 owing to a rotation of the ligand to form a site that we designate site 1A in the F728A mutant, as discussed below.

**Figure 2:**
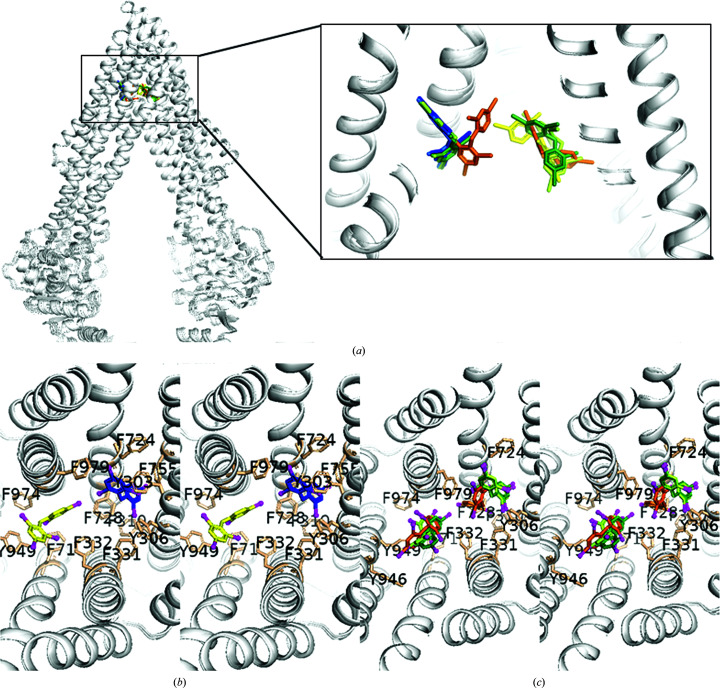
Localization of BDE-100 in the DBP and the role of aromatic residues. (*a*) Superposition of BDE-100 for all six mutants (C952A parent, F979A, F724A, F728A, Y303A and Y306A) shown for the inward-facing conformation of Pgp, depicting the relative localization within the DBP. (*b*, *c*) Stereoviews of close-ups of the alternate BDE-100 binding sites. (*b*) shows composite structures of the single-site binders C952A (which was indistinguishable from F979A) and F724A. (*c*) shows composites of the two-site binders Y303A, Y306A and F728A. The ligand in C952A is colored dark blue, that in F724A is colored yellow, that in Y303A is colored light green, that in Y306A is colored dark green and that in F728A is colored orange. Aromatic side chains in the DBP that play a role in binding in at least one site (≤4.2 Å from the ligand) are shown as sticks colored tan and are labeled.

73% (8/11) of the residues that form site 1 are aromatic in character. For sites 2 and 3, the percentages of aromatic residues contributing to ligand binding are 67% (4/6) and 56% (5/9), respectively (Fig. 2 ▸). The ability of alternate aromatic residues of sites 2 and 3 to take part in ligand binding when the primary binding site was mutated reveals that aromaticity is important for polyspecificity. The residues forming sites 1–3, located on TM5, TM6, TM11 and TM12, have previously been shown to bind both substrates and inhibitors (Alam *et al.*, 2019 ▸; Szewczyk *et al.*, 2015 ▸; Wang *et al.*, 2003 ▸; Zhou, 2008 ▸). This suggests that the primary ligand-interacting residues in Pgp do not by themselves in a static manner distinguish between the inhibitory or stimulatory effects of the ligand on ATPase activity. Occupancy refinements showed good BDE-100 occupancy in site 1 for the C952A control, Y303A, Y306A and F979A mutants (0.64–0.68) that were comparable to the occupancies we achieved in our refinements of the published structure with PDB code 4xwk (0.66) using the deposited structure factors (Table 2 ▸; see Section 2 for details). The novel site 3 binding site in the F724A mutant refined to a considerably lower occupancy of ∼0.4 compared with the occupancy of site 1 in the C952A control, Y303A, Y306A and F979A mutants. The ligand occupancies for site 1 were in the range 0.5–0.6.

**Figure 3:**
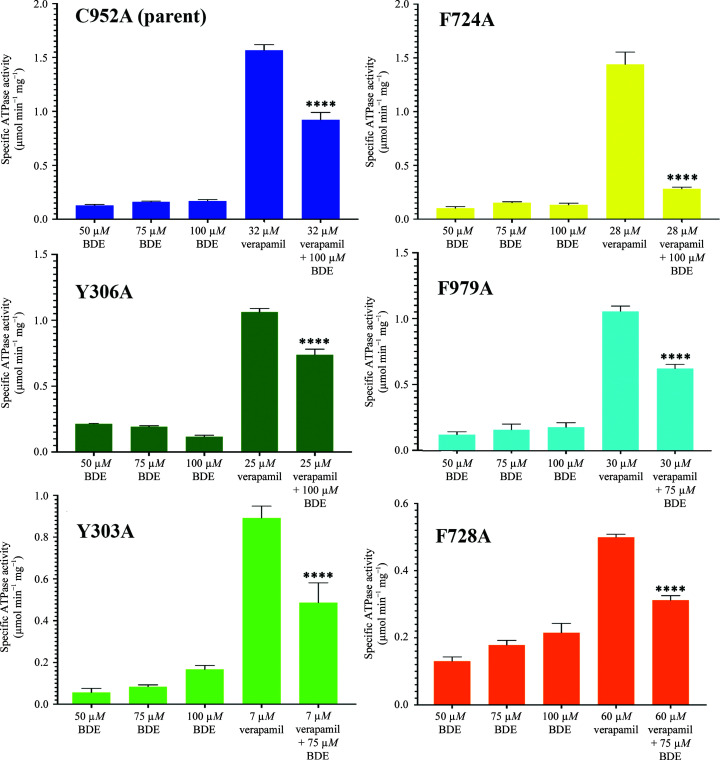
The effect of BDE-100 on the verapamil-stimulated ATPase activity of the six Pgp mutations. The effect of BDE-100 in the absence of verapamil on each mutant was determined at 50, 75 and 100 µ*M*. The EC_50_ of verapamil-stimulated ATPase activity of each mutant was pre-determined (Supplementary Fig. S4) and is shown here. The level of BDE-100 inhibition of the verapamil-stimulated ATPase EC_50_ for each mutant is also shown. *P* values were were determined from a two-tailed unequal variance test: ****, *p* ≤ 0.0001; ***, *p* ≤0.0001–0.001; *, *p* ≤0.05–0.01. The scale of each graph is represented to enhance the differences of verapamil concentration on ATPase for the given mutant, particularly since one mutant, F728A, exhibited a considerably lower *V*_max_ compared with the other mutants.

**Figure 4:**
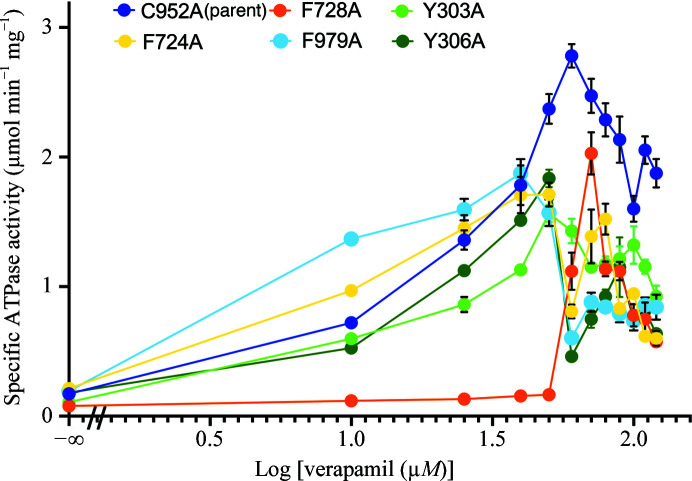
Dose–response curve of verapamil-stimulated ATPase activity. Curves for each different mutant are colored according to the legend of the figure. The F728A mutant exhibited the highest EC_50_ of 60 µ*M*, followed by C952A (32 µ*M*), F979A (30 µ*M*), F724A (28 µ*M*), Y306A (25 µ*M*) and Y303A (7.3 µ*M*). All measurements were determined with seven independent measurements (*n* = 7).

Previous mutagenesis studies demonstrated that there was no loss of multidrug resistance in a cell-survival assay for nearly every Phe-to-Ala point mutation examined in human Pgp (Loo & Clarke, 1993 ▸). Not surprisingly, the corresponding mutations of mouse Pgp used in this work were each still competent in verapamil-stimulated ATP hydrolysis, albeit with differences in ATPase kinetics. Increasing concentrations of BDE-100 alone did not increase the basal ATPase activity for wild-type Pgp (Nicklisch *et al.*, 2016 ▸), the C952A control or any of the five aromatic replacement mutations (Fig. 3 ▸ and Supplementary Fig. S5). These results suggested that BDE-100 itself is a poorly transported substrate of Pgp. In contrast, BDE-100 exhibited a significant inhibitory effect on verapamil-stimulated ATPase activity for all five mutants at the verapamil EC_50_ determined for each of the five mutants and the control C952A (Fig. 4 ▸).

**Figure 5:**
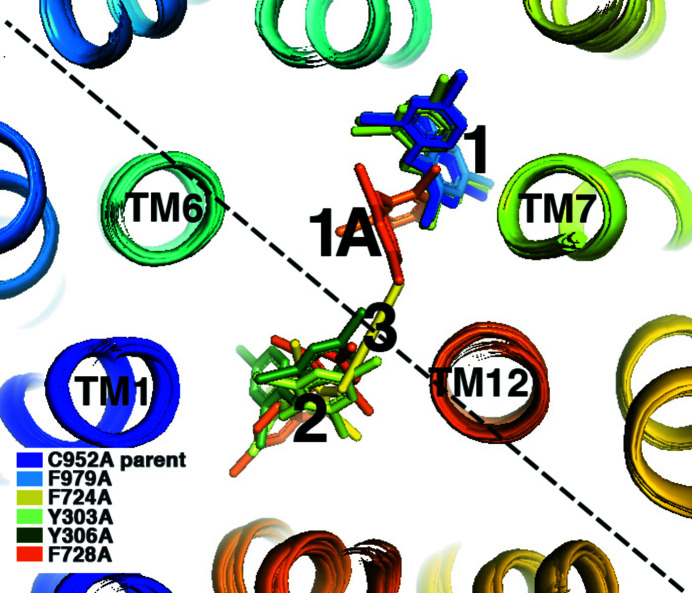
Composite view of the DBP showing four distinct BDE-100 binding sites depending on the mutation of Pgp. Transmembrane (TM) α-helices are shown as ribbons and are colored from the N-terminus to the C-terminus (blue to orange). Two critical TM helices known to bind multiple different ligands, TM6 and TM12, are labeled. Dual DBE-100 occupancy for the Y303A, Y306A and F728A mutants was apparent, in which the two binding sites are in opposing ‘halves’ of the pseudosymmetric DBP. The F724A mutant structure was distinct from all others in that it contains a single BDE-100 bound in a novel site that lies on the axis of pseudosymmetry (dotted line), which we designate site 3. The rationale for the site nomenclature is as follows. Site 1 is the canonical site previously discovered by Nicklisch *et al.* (2016). Some structures with site 1 or site 1A occupancy also had a second site occupancy, which we designate site 2. Site 3 is the distinct novel single-site binding unique to F724A.

Taken together, polyspecific ligand recognition by Pgp appears to comprise a high degree of plasticity in the capacity for ligand binding to the DBP (Fig. 5 ▸). Loss of a direct aromatic contact to the ligand did not block ligand binding in any of the five mutants compared with the C952A control, but instead the following were observed: (i) binding to site 1 was precisely retained for three mutants (Y303A, Y306A and F979A), (ii) detectable localization of ligand binding to a second site (site 2) was observed for three mutants (Y303A, Y306A and F728A), (iii) one mutant (F728A) also exhibited occupancy near site 1 in which the ligand was rotated about TM7 by ∼75° towards the axis of pseudosymmetry that we refer to as site 1A, and (iv) the F724A mutant only showed detectable binding to site 3, which is located directly on the axis of pseudosymmetry of the transporter.

Phe974 in particular appears to make important contributions to the compensatory ligand-recognition mechanism. More specifically, close inspection of the 2*mF*_o_ − *DF*_c_ electron density in the structures of the C952A control and F979A mutants, as well as the previously published Pgp–BDE-100 structure (PDB entry 4xwk), revealed that the Phe974 side chain is highly disordered when BDE-100 is not present in any other site besides the canonical BDE-100 site 1 (Fig. 1 ▸). In these three structures the side chain refined to its most favored rotameric preference (76% in the rotamer library) given the absence of electron density to support any specific rotamer. This preferred side-chain position, however, overlaps considerably with anomalous difference electron density of BDE-100 bound to alternate/additional sites in the structures of the Y303A, Y306A, F724A and F728A mutants when viewed using a superposition of all structures and electron-density maps. Furthermore, a refined position of a rare rotamer of Phe974 that points away from the pseudosymmetric axis of the DBP was preferred in all structures with BDE-100 in site 2, had visible coverage by 2*mF*_o_ − *DF*_c_ electron density and made direct contact with the ligand. We conclude that Phe974 undergoes an induced fit-like disorder-to-order transition to support the binding of BDE-100 in sites 2 and 3, and this residue is therefore important for polyspecific recognition of the BDE-100 ligand when the primary binding site (site 1) is challenged by mutagenesis.

Phe724 and Phe728 (both on TM7) appear to be important for maintaining the integrity of the canonical BDE-100 site 1, since the mutation of either residue resulted in ligand shifts away from this site. The F728A mutation resulted in an ∼75° rotation of the ligand around TM7 at the pivot point of Ala728 to site 1A that is closer to the pseudosymmetric axis and maintains van der Waals contact between the ligand and TM7 [Figs. 1 ▸(*d*) and Fig. 5 ▸]. Despite the rotation of the ligand in the F728A mutant structure, there was sufficient space in the DBP to accommodate the binding of another ligand molecule in site 2. The F724A mutation resulted in a greater shift of binding of a single ligand molecule in site 3 that lies directly on the pseudosymmetric axis of the transporter. Site 3 overlaps in 3D space with sites 1A and site 2 in the other structures, and there does not appear to be sufficient space to accommodate more than one ligand in the F724A structure.

Ligands in sites 1A, 2 and 3 generally exhibited greater disorder compared with those in site 1, as shown by higher *B* factors in refinement and some smearing of the anomalous electron density. Nevertheless, the pattern of anomalous density was highly consistent between multiple crystals of each mutant (Y303A, *n* = 8; Y306A, *n* = 5; F724A, *n* = 4; F728A, *n* = 8, F979A, *n* = 3; C952A parent, *n* = 3). It is tempting to speculate that some disorder of the ligand is tolerated in the alternate sites as a form of polyspecificity. In other words, very tight, ordered binding may not be essential for recognition by Pgp (and presumably transport), and may slow transport rates if the Gibbs binding free energy becomes highly favorable for certain substrates, particularly if hydrogen bonding is involved (Seelig & Landwojtowicz, 2000 ▸).

The differences between the Y303A and Y306A mutants present a puzzling element of their roles in polyspecificity. The pattern of anomalous density for eight crystals of the Y303A mutant and five crystals of the Y306A mutant clearly shows a slightly different binding pose of BDE-100 in their respective sites 2 (not shown), yet Tyr303 and Tyr306 are involved in site 1 formation and are as far from site 2 as any site 1 residue. Furthermore, we detected no significant differences in the binding pose of BDE-100 in site 1 between the Y303A, Y306A, C952A or F979A mutants. One possibility is that long-range communication, or potentially quantum considerations, between site 1 binding and site 2 occurs but is not detectable given the limits of our data.

## 4. Conclusions   

Compensatory ligand recognition appears to contribute an important component to the mechanism of polyspecificity in MDR1/Pgp. In essence, polyspecificity is the opposite of ‘lock-and-key’ recognition in which, in this case, the lock (Pgp DBP) appears to recognize different keys (substrates) even when the shape of the lock is changed slightly at specific locations. For Pgp, the loss of one of several individual critical aromatic contacts in the DBP by mutagenesis to alanine allowed a shift in the binding of the BDE-100 ligand while still preserving the ligand’s known function of ATPase inhibition. Since aromatic residues are a dominant component of both the canonical and the novel ligand sites, we conclude that aromaticity itself is fundamental to the mechanism of polyspecificity. A slight perturbation of the canonical site allowed a second copy of BDE-100 to bind to the DBP in some cases, and therefore stoichiometry does not appear to be a stringent requirement for polyspecificity. In our structures, the transporter is essentially in the same conformation regardless of the amino-acid mutation, allowing us to conclude that the binding shifts were not owing to differences in the overall protein conformation. It would thus seem that the DBP of Pgp in the conformation that we characterized by XRC must have a relatively flat energy landscape, at least with respect to BDE-100, that allows the ligand to move to new sites if the primary binding site is perturbed. The compensatory nature of polyspecific ligand recognition would also seem to make it very challenging to modify any chemotherapeutic drug substrate that would allow it to evade efflux by Pgp altogether.

## Supplementary Material

Supporting figures and legends. DOI: 10.1107/S2052252520005709/jt5047sup1.pdf

PDB reference: P-glycoprotein, C952A mutant, complex with BDE-100, 6unj

PDB reference: C952A/F979A mutant, complex with BDE-100, 6ujp

PDB reference: C952A/F724A mutant, complex with BDE-100, 6ujr

PDB reference: C952A/F728A mutant, complex with BDE-100, 6ujs

PDB reference: C952A/Y303A mutant, complex with BDE-100, 6ujt

PDB reference: C952A/Y306A mutant, complex with BDE-100, 6ujw

## Figures and Tables

**Table 1:** Data-collection and refinement statistics Values in parentheses are for the outer shell.

	C952A	Y303A	Y306A	F724A	F728A	F979A
Data collection						
*a* (Å)	87.57	91.12	90.96	90.40	91.50	89.05
*b* (Å)	137.54	138.65	138.84	138.03	138.25	138.13
*c* (Å)	184.51	195.40	196.76	193.89	196.13	188.25
α = β = γ (°)	90	90	90	90	90	90
Resolution range (Å)	30.0–3.98 (4.05–3.98)	30.0–4.17 (4.24–4.17)	30.0–4.15 (4.22–4.15)	30.0–4.10 (4.17–4.10)	30.0–4.17 (4.24–4.17)	30.0–3.98 (4.05–3.98)
*R*_merge_ (%)	7.1 (70.8)	6.5 (66.9)	5.9 (74.1)	7.6 (71.0)	6.2 (73.8)	8.0 (66.7)
*R*_p.i.m._ (%)	2.5 (40.5)	1.8 (40.6)	2.0 (46.0)	1.9 (37.6)	2.6 (41.7)	2.8 (44.9)
Unique reflections	19668	19030	19407	19601	19111	20499
Mean *I*/σ(*I*)	22 (1.2)	28 (1.0)	30 (1.1)	29 (1.2)	23 (1.1)	24 (1.3)
Completeness (%)	99.9 (99.9)	100 (100)	100 (100)	100 (100)	100 (100)	99.9 (98.0)
Wilson *B* factor (Å^2^)	165.6	205.2	198.3	195.6	188.1	170.2
Refinement						
Resolution range (Å)	30.0–3.98 (4.04–3.98)	30.0–4.17 (4.24–4.17)	30.0–4.15 (4.21–4.15)	30.0–4.10 (4.16–4.10)	30.0–4.17 (4.23–4.17)	30.0–3.98 (4.04–3.98)
*R*_work_/*R*_free_	0.245/0.264	0.250/0.278	0.251/0.286	0.271/0.295	0.250/0.289	0.239/0.263
*R*_free_, high res.	0.307	0.322	0.385	0.366	0.277	0.349
Phase error (°)	28.2	29.8	31.4	31.6	30.2	28.7
R.m.s. deviations						
Bond lengths (Å)	0.003	0.003	0.002	0.003	0.002	0.003
Bond angles (°)	0.661	0.727	0.523	0.639	0.501	0.580
Average *B* factor (Å^2^)						
Protein	190.9	230.2	232.4	217.5	210.1	197.4
BDE-100	208.7	262.6	256.1	238.4	249.4	218.2
Ramachandran statistics						
Favored (%)	94.57	94.97	95.25	95.76	95.59	95.84
Outliers (%)	0.51	0.34	0.17	0.25	0.17	0.17
Rotamer outliers (%)	0.51	0.62	3.81	0.51	2.88	3.71
C^β^ deviations	0	0	0	0	0	0

**Table 2:** Occupancy values of BDE-100 in site 1, site 2 and site 3 in the C952A, F979A, F724A, F728A, Y303A and Y306A mutants

	Occupancy (%)		
	Site 1	Site 2	Site 3
C952A	67		
F979A	65		
F724A			35
F728A	53†	48	
Y303A	64	56	
Y306A	68	49	
PDB entry 4xwk	66		

† Designated site 1A.
