# Supplementary material for: Structural definition of polyspecific compensatory ligand recognition by P-glycoprotein
Source: IUCrJ. 2020 Jun 6;7(Pt 4):663–72. doi: 10.1107/S2052252520005709 (PMC7340268; doi:10.1107/S2052252520005709)
Supplement: Supplementary file 1 [file m-07-00663-sup1.pdf]

# IUCrJ

**Volume 7 (2020)**


**Supporting information for article:**

## Structural definition of polyspecific compensatory ligand recognition by P-glycoprotein

**Christina A. Le, Daniel S. Harvey and Stephen G. Aller**

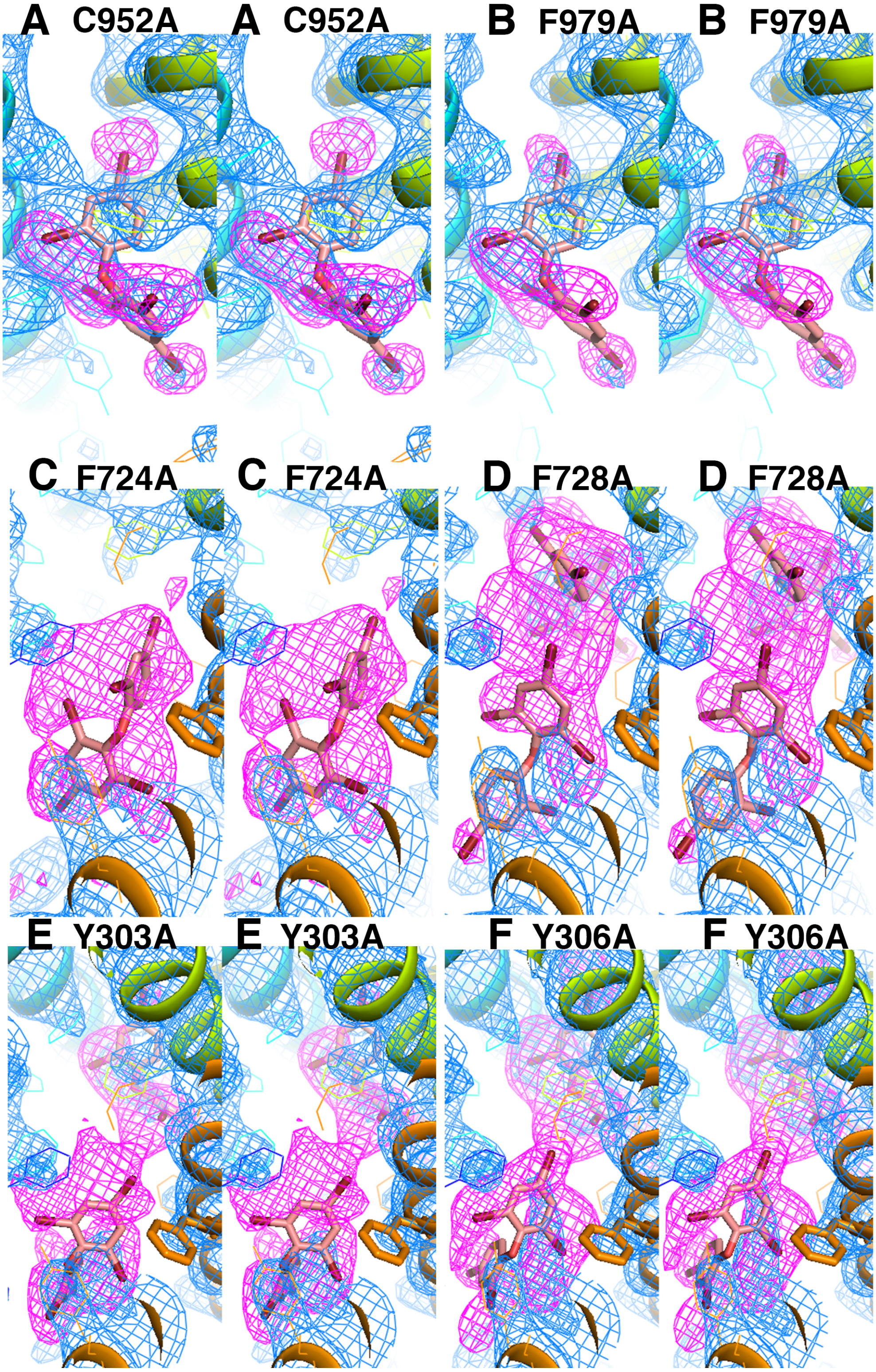


**Figure S1** Electron density of six Pgp mutants and localization of BDE-100 by x-ray crystallography. Stereo views are from within the membrane roughly parallel to the membrane plane. Blue mesh represents $2mF_o$-$DFc$ density (B-factor sharpened by 50-75 $A^2$) contoured to 1.0σ. Magenta mesh represents anomalous difference fourier electron density from the bromine atoms of BDE-100 using x-ray data collected at the bromine anomalous absorption peak (13.48 keV). Contour levels as follows: 5.5σ for C952A, 5.0σ for F979A, 4.0σ for F724A, 3.8σ for F728A, 4.2σ for Y303A and Y306A. BDE-100 in each structure is shown in stick representation with bromine atoms colored red.

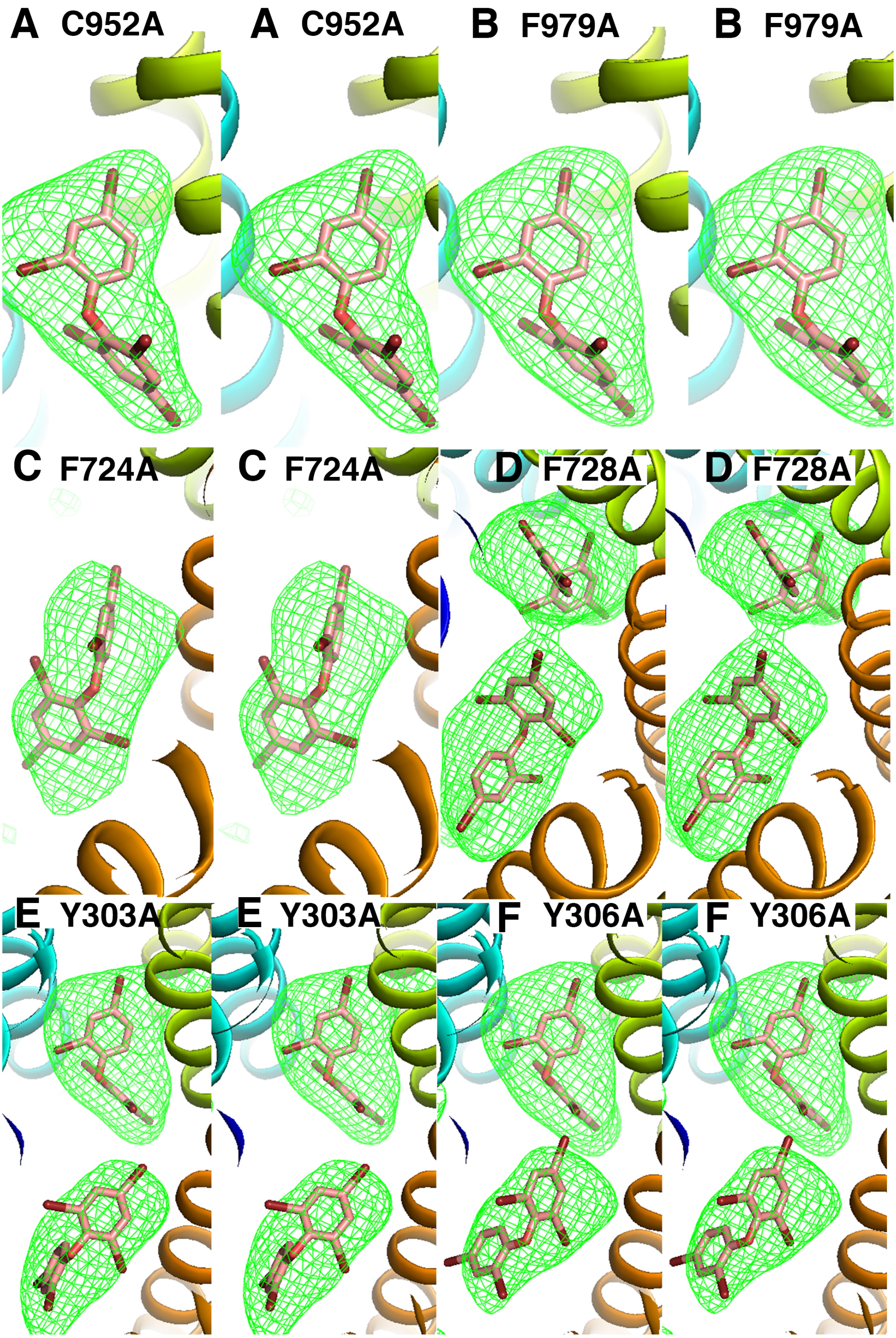


**Figure S2** BDE-100 omit electron density maps (Fo-Fc) for the six mutations of Pgp (wall eyed stereo). Omit maps are shown in green (contour levels of 3σ for C952A and 979A, 2.5σ for Y303A and Y306A, and 2.0σ for F724A and F728A).

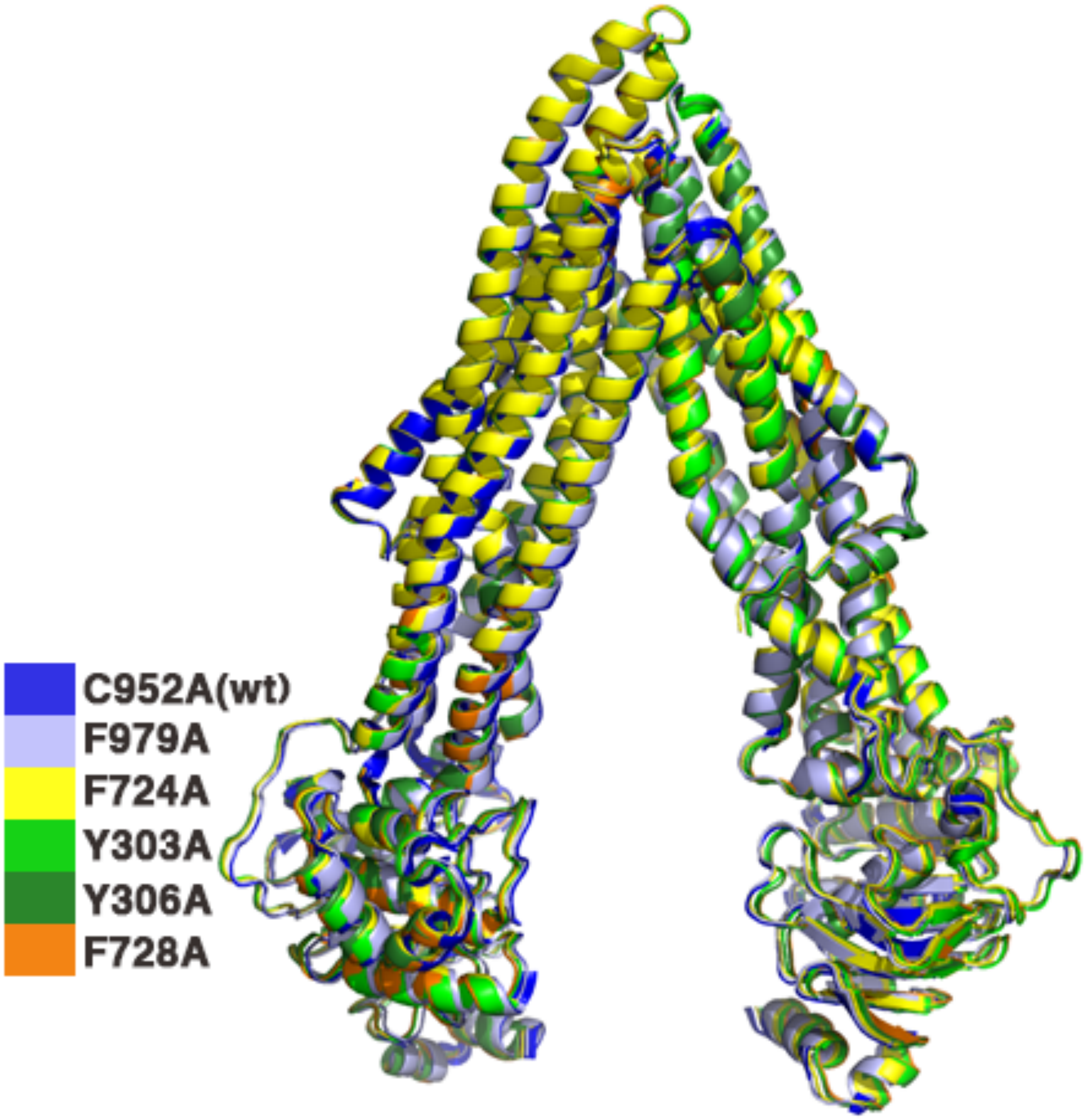


**Figure S3** Superposition of all six crystal structures of single site mutations of Pgp determined in this work. C952A is shown in dark blue, F979A in light blue, F724A in yellow, Y303A is shown in green, Y306A dark green, and F728A in orange. Although there were minute changes in some of the TM helices and nucleotide binding domains, the global structures are nearly identical.

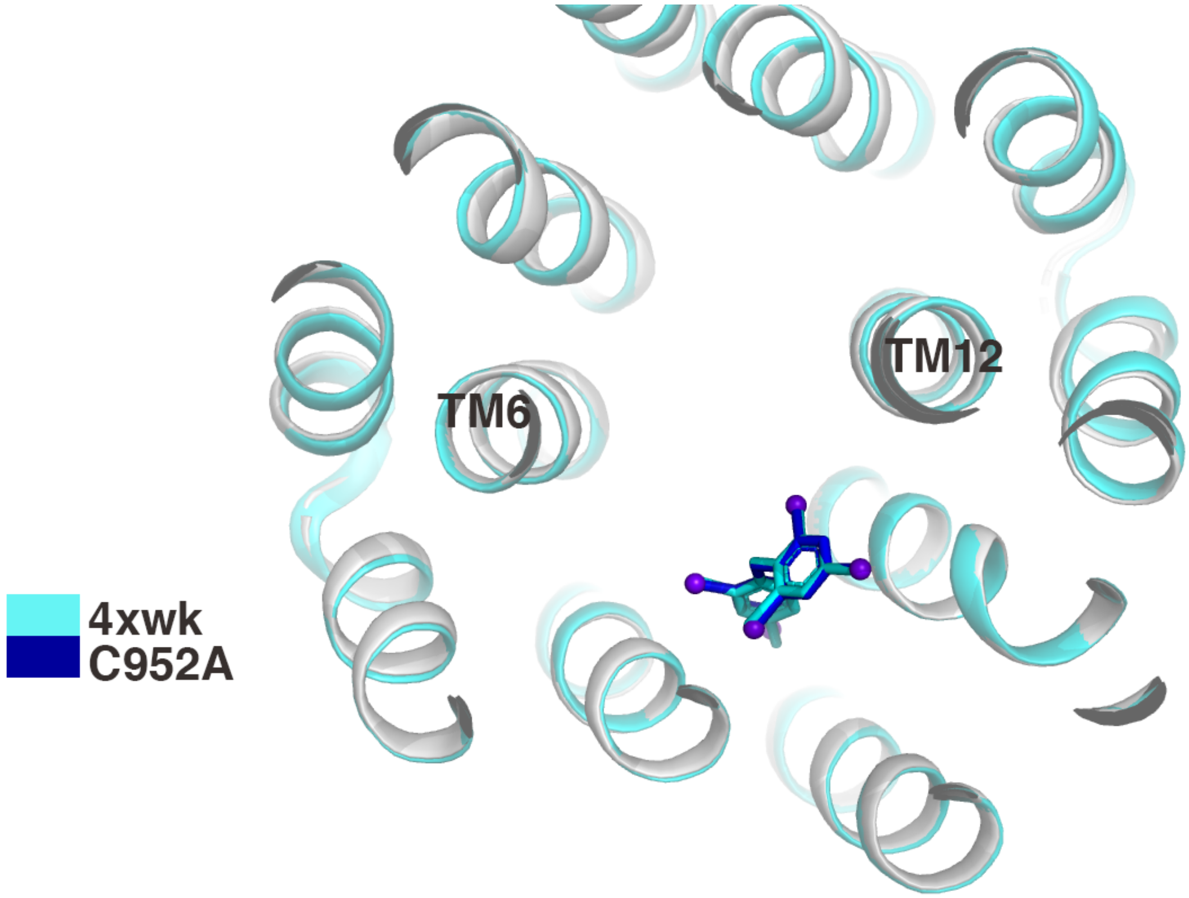


**Figure S4** Alignment of C952A and 4xwk. C952A BDE100 is shown in dark blue while the structure is in grey and 4xwk is shown in light blue. Although there were minute changes in some of the TM helices and nucleotide binding domains, the position of BDE100 between both structures are identical.

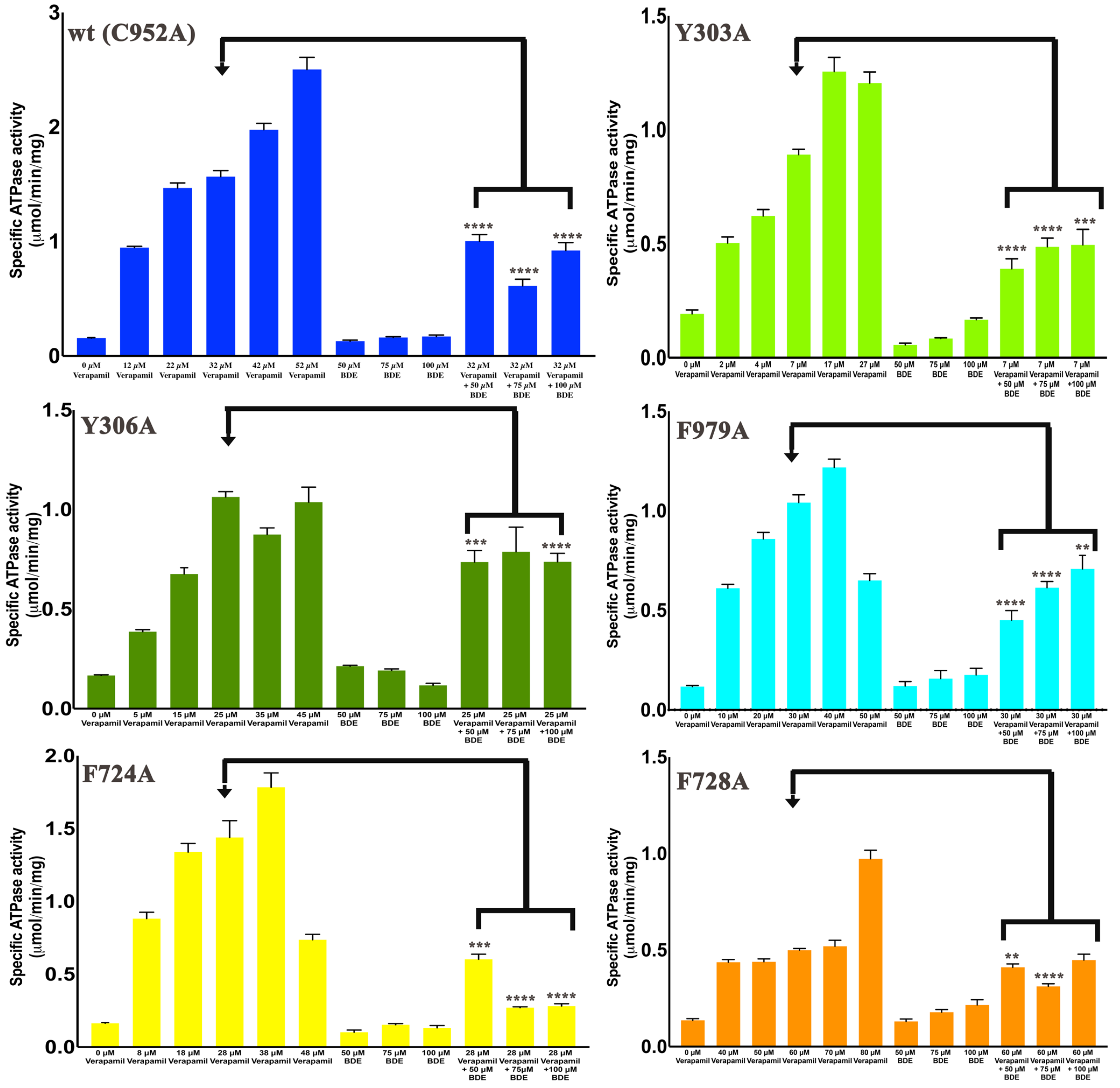


**Figure S5** Dose response curve of Verapamil stimulated ATPase activity for the six site-specific mutations of Pgp. Verapamil concentrations clustered around the pre-determined $EC_{50}$ for Verapamil (Figure 4) were chosen to yield a more finely sampled dose response for this figure. BDE-100 alone had little effect on Pgp ATPase activity. Inhibitory effects of BDE-100 at 50 µM, 75 µM and 100 µM on the Verapamil-$EC_{50}$ specific to each mutant were determined. All measurements were determined with seven independent measurements (n=7).

| PDB code | phase error (˚) | resolution (Å) |
|---|---|---|
| 4M1M | 27.5 | 3.80 |
| 6UJN | 28.2 | 3.89 |
| 6UJP | 28.7 | 3.89 |
| 6UJT | 29.9 | 4.17 |
| 6UJS | 30.2 | 4.17 |
| 5KO2 | 30.5 | 3.30 |
| 5KOY | 31.0 | 3.85 |
| 6UJW | 31.5 | 4.15 |
| 6UJR | 31.6 | 4.10 |
| 4M2T | 32.1 | 4.20 |
| 4M2S | 32.7 | 4.30 |
| 5KPD | 34.1 | 3.35 |
| 4Q9I | 34.1 | 3.78 |
| 4KSD | 34.2 | 4.10 |
| 4Q9J | 34.3 | 3.60 |
| 4XWK | 34.5 | 3.50 |
| 5KPI | 35.2 | 4.00 |
| 4Q9L | 35.5 | 3.80 |
| 4Q9H | 35.7 | 3.40 |
| 4KSC | 35.9 | 4.00 |
| 4Q9K | 36.2 | 3.80 |
| 4KSB | 38.4 | 3.80 |
| 5KPJ | 40.4 | 3.50 |

**Table S1**. Phase error statistic and resolution shown for all published x-ray crystal structures of mouse P-glycoprotein.
